# Application of FT-IR spectroscopy and chemometric technique for the identification of three different parts of *Camellia nitidissima* and discrimination of its authenticated product

**DOI:** 10.3389/fphar.2022.931203

**Published:** 2022-09-27

**Authors:** Wan Yin Tew, Chen Ying, Zhang Wujun, Liu Baocai, Tiem Leong Yoon, Mun Fei Yam, Chen Jingying

**Affiliations:** ^1^ Research Center for Medicinal Plant, Institute of Agricultural Bio-resource, Fujian Academy of Agricultural Sciences, Fuzhou, China; ^2^ School of Pharmaceutical Sciences, Universiti Sains Malaysia, Gelugor, Malaysia; ^3^ School of Chinese MateriaMedica, Beijing University of Chinese Medicine, Beijing, China; ^4^ School of Physics, Universiti Sains Malaysia, Gelugor, Malaysia; ^5^ College of Pharmacy, Fujian University of Traditional Chinese Medicine, Fuzhou, China

**Keywords:** golden camellia, Fourier transform infrared spectroscopy, second-derivative infrared spectra, two-dimensional correlation infrared spectra, principal component analysis, orthogonal partial least square discriminant analysis

## Abstract

*Camellia nitidissima* C.W. Chi is a golden camellia recognized in Chinese herbology and widely used as tea and essential oil in Chinese communities. Due to its diverse pharmacological properties, it can be used to treat various diseases. However, unethical sellers adulterated the flower with other parts of *Camellia nitidissima* in their product. This study used an integrated tri-step infrared spectroscopy method and a chemometric approach to distinguish *C. nitidissima*’s flowers, leaves, and seeds. The three different parts of *C. nitidissima* were well distinguished using Fourier transform infrared spectroscopy (FT-IR), second-derivative infrared (SD-IR) spectra, and two-dimensional correlation infrared (2D-IR) spectra. The FT-IR and SD-IR spectra of the samples were subjected to principal component analysis (PCA), PCA-class, and orthogonal partial least square discriminant analysis (OPLS-DA) for classification and discrimination studies. The three parts of *C. nitidissima* were well separated and discriminated by PCA and OPLS-DA. The PCA-class model’s sensitivity, accuracy, and specificity were all >94%, indicating that PCA-class is the good model. In addition, the RMSEE, RMSEP, and RMSECV values for the OPLS-DA model were low, and the model’s sensitivity, accuracy, and specificity were all 100%, showing that it is the excellent one. In addition, PCA-class and OPLS-DA obtained scores of 27/32 and 26/32, respectively, for detecting adulterated and other TCM reference flower samples from *C. nitidissima*. Combining an infrared spectroscopic method with a chemometric approach proved that it is possible to differentiate distinct sections of *C. nitidissima* and discriminate adulterated samples of *C.nitidissima* flower.

## Introduction

*Camellia*, also known as “cha-hua” in Chinese, is the largest genus of flowering plants in the Theaceae family. It consists of nearly 200 known species and is widely dispersed throughout China. They are ubiquitous in China and southeastern and eastern Asia, where over 80% of these species are present (Vijayan et al., 2009). Their flowers are usually red, purple, pink, or white. Additionally, they have less yellow blossoms, making them the most sought-after variation of this plant’s flower. Camellia with deep yellow blossoms is also referred to as “golden camellia” and was discovered in Guangxi, China, in 1965 by Hu, who named it *Theopsis chrysantha* (Hu, 1965). The taxon was then renamed “*Camellia*” and subsequently “*Camellia chrysantha*” (Tuyuma, 1975).

It is believed that golden camellia has a variety of pharmacologically active compounds, including polysaccharides, saponins, polyphenols, and flavonoids, which are frequently used by the Chinese to make both tea and traditional medicines (Do et al., 2019). According to a recent study, golden camellia contains more phenolic compounds than other species of the same genus, such as green tea (*Camellia sinensis*) (Song et al., 2011). In golden camellia, polyphenolic compounds such as quercetin, kaempferol derivatives, and apigenin have been identified, but only in trace amounts in normal tea leaves (Lin et al., 2013; Wang et al., 2017). These factors enhanced the therapeutic value and pharmacological potential of golden camellias.

*Camellia nitidissima* C.W. Chi is a rare and well-known ornamental camellia species that is listed in the Compendium of Materia Medica, Ben Cao Gang Mu (*本草纲目*). It was used to treat nephritis, hepatitis, jaundice, urinary tract infections, dysentery, hypertension, diarrhea, liver cirrhosis, sores, and irregular menstruation (He et al., 2017). Flowers and leaves of *C. nitidissima* have been commercially cultivated as a new source of tea leaves. Its seeds have been used to produce essential oils, and both flowers and leaves are extensively utilized by Chinese communities (Wang et al., 2018). In addition to offering functional benefits to the community *via* non-pharmacological and lifestyle adaptations, it has been discovered to exert a wide range of pharmacological effects. It can be an useful agent with anticancer, antioxidant, hypolipidemic, antidiabetic, antiallergic, antibacterial, and anxiolytic properties (He et al., 2017; An et al., 2020). Previous research study has shown that *C.nitidissima* has neuroprotective benefits through a synergistic interaction between antioxidant and neurotrophic signaling pathways (An et al., 2020). Despite the growing number of research studies describing various pharmacological activities of *C. nitidissima*, there are no data on its chemical fingerprinting or the chemical composition of its leaves, flowers, and seeds. The plant’s chemical composition may vary depending on the species, location, age, harvesting system, and drying method. Different parts of a similar plant may contain significantly different components, especially the major ones (Heinrich, 2015).

Several approaches, such as the widely employed chromatography techniques in laboratory research, can be utilized to examine chemical compositions and provide a comprehensive overview of medicinal plants (Yang et al., 2013; Kitanov et al., 2015). However, chromatography necessitates a difficult sample preparation method, and it is time-consuming to obtain a complete analysis (Peerapattana et al., 2015). Based on the current market value, 1 kg of *C. nitidissima* flower will cost around 2,000–3,000 Chinese yuan (RMB), whereas *C. nitidissima* leaf costs only 200–400 RMB. Due to the considerably larger market price of *C. nitidissima* flower, it is feasible for unscrupulous vendors to adulterate or mix the flower with the leaves in their beverage or product. Chromatography may make it difficult to discern between leaves and flowers with similar phyto-compound profiles.

Fourier-transform infrared spectroscopy (FT-IR) is another widely practiced method for obtaining chemical fingerprinting. In recent years, numerous studies have been conducted about the application of FT-IR in medicinal plants in various aspects (Liu et al., 2010). Compared to chromatography, it needs minimum sample preparation and can identify multiple components in a single study (Moros et al., 2010; Smith, 2011). FT-IR spectroscopy offers a broad scope for studying medicinal plants in conjunction with chemometric analysis. Chemometrics, a multidimensional statistical analysis methodology that retrieves information from data *via* the application of mathematics and statistics, will obtain valuable chemical information from the original spectral data *via* unsupervised (PCA) and supervised classification (PLS and OPLS) methods (Rohman et al., 2019). Our study categorized the different parts of *C. nitidissima* samples using FT-IR with principal component analysis (PCA), PCA-class, and OPLS-DA analysis methods. PCA-class and OPLS-DA were also used to discriminate the adulterated samples and TCM flowers from *C. nitidissima*.

### Methodology

#### Apparatus

A spectrum two Fourier-transform infrared (FT-IR) spectrometer (PerkinElmer, United States) characterized the samples. A programmable temperature controller (FTIR Solution, Malaysia) was utilized for thermal perturbation.

### Samples and materials

A total of 121 *Camellia nitidissima* samples were provided by Prof. Chen Jingying (Fujian Academy of Agricultural Sciences), including 34 flowers, 80 leaves, and 7 seed samples. All samples were collected from Guangxi and Fujian (Zhangping, Yongfu, and Shanghang provinces) between 2016 and 2017. In addition, 22 *Camellia nitidissima* adulterated samples from the market and 10 TCM reference flower samples from the National Institutes for Food and Drug Control, China, were included for OPLS-DA and PCA-class discrimination and classification studies, respectively. Potassium bromide (KBr) was purchased from Merck (Germany) and used as the background for FT-IR analysis (Merck, Germany).

### Procedure for FT-IR spectral acquisition

Before the experiment, KBr was dried overnight at 120°C to remove all traces of moisture. Each sample was pulverized and mixed with crystalline KBr. Subsequently, the mixtures were ground and compressed into tablets. The spectra were recorded from 4000 to 400 cm^−1^ (mid-infrared region) at a resolution of 4 cm^−1^ with a 1 cm^−1^ interval. Each spectrum was calculated from 16 co-added scans to minimize the signal-to-noise ratio (SNR) and improve the spectral quality. The raw FT-IR spectrum was processed using Spectrum 10.5.3 (PerkinElmer, United States). The FT-IR spectrum was accepted when the achieved transmission was 60% or higher (the lowest peak located within 30–10% of transmission). Alternately, the test was repeated with KBr or sample addition until at least 60% transmission was achieved. The pellets were inserted into a sample holder equipped with a programmed heating jacket (FTIR Solution, Malaysia) to acquire the 2D correlation infrared spectrum. The sample holder was then heated, and the spectra were collected at different temperatures ranging from 20°C to 120°C at a 10°C interval.

### Data processing for second-derivative and two-dimensional correlation spectrum

After obtaining the FT-IR spectra, they were converted to a second-derivative spectrum with 13 data points for slope calculation. Moreover, the FT-IR spectrum also undergoes baseline correction and smoothing before performing arithmetic correction. The 2D-IR correlation spectra were calculated and obtained *via* treating the spectra with 2D-IR correlation analysis software developed by Tsinghua University (Beijing, China).

### Chemometric analysis

In the preliminary phase, unsupervised pattern recognition techniques known as principal component analysis (PCA) were utilized to examine variations in the FT-IR spectral characteristics of various parts of *C. nitidissima*, adulterated, and TCM reference flower samples. After PCA analysis, PCA-class and orthogonal partial least square discriminant analysis (OPLS-DA) were adapted for the discrimination study. The samples were randomly divided into calibration and validation sets. When conducting PCA-class and OPLS-DA model, 60% of the spectra from different parts were considered a calibration set, and the remaining spectra (40% of *C. nitidissima*, adulterated, and TCM reference flower samples) were considered a validation set. The specificity, accuracy, and sensitivity of PCA-class and OPLS-DA for differentiating the validation set were determined. For the OPLS-DA study, R^2^Y(presentation of the variation of the calibration set-Y, explained by the model), Q^2^Y (presentation of the variation of the calibration set-Y, predicted by the model according to cross-validation), root mean-squared error of estimation (RMSEE), root mean-squared error of cross-validation (RMSECV), and root mean-squared error of prediction (RMSEP) were calculated. The cross-validation method with seven cancellation groups was conducted to verify the robustness of the model. The permutation test was conducted as internal validation, with 100 permutations predetermined. In addition, three parameters, namely, sensitivity, specificity, and accuracy, were computed to evaluate the performance of the calibration model. All OPLS-DA, PCA, and PCA-class models were established using SIMCA 14.1 (Umetrics, Sweden).

## Results and discussion

### Differentiation by FT-IR spectra

**FIGURE 1:**
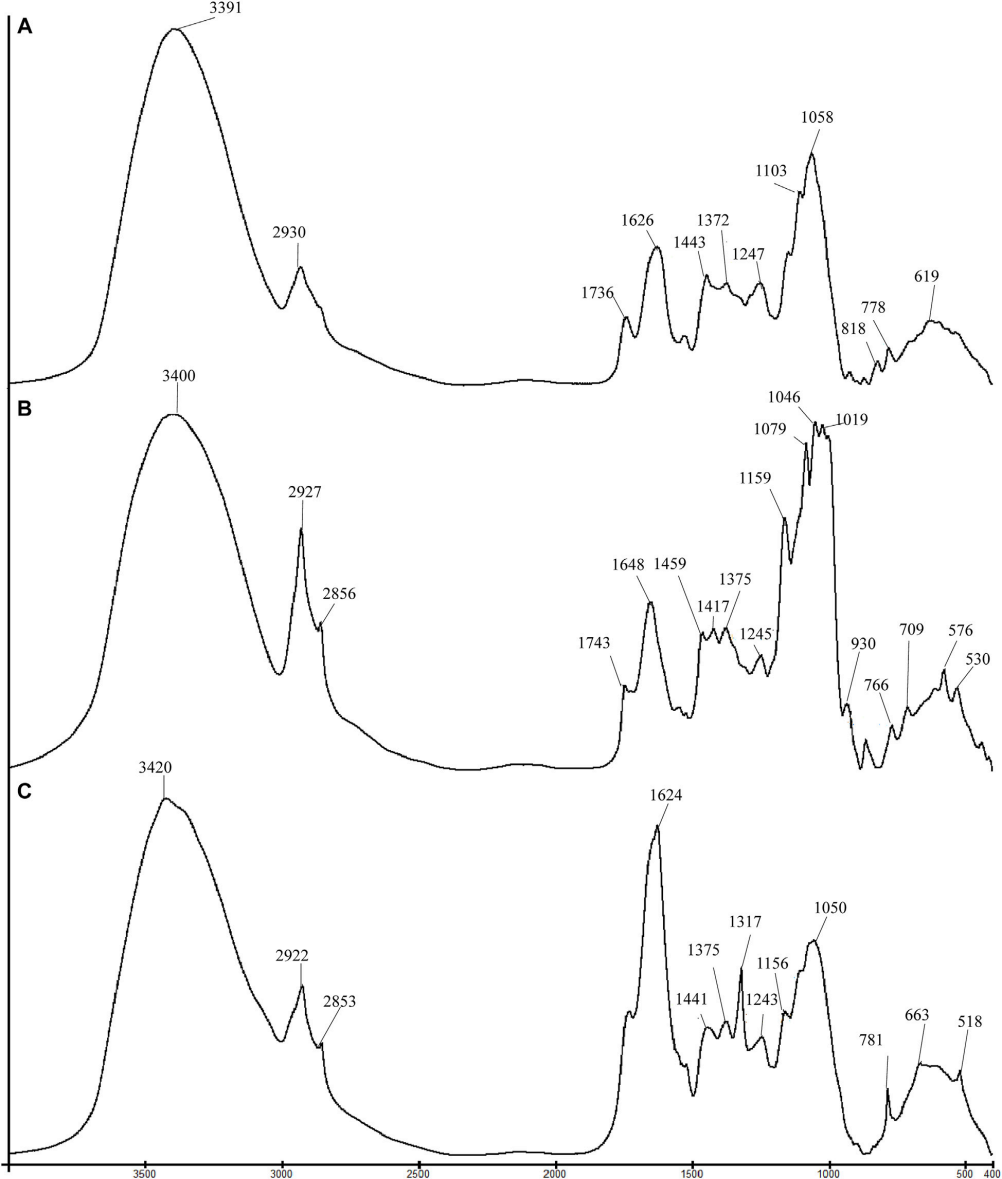
Comparison of FT-IR spectra for different parts of *Camellia nitidissima*: **(A)** flower, **(B)** seed, and **(C)** leaf.

Tri-step infrared spectroscopy is an analytical method explicitly applied for analyzing complex systems. It comprises conventional FT-IR, second-derivative infrared spectroscopy (SD-IR), and two-dimensional correlation infrared spectroscopy (2D-IR) (Liu et al., 2018). FT-IR spectroscopy was utilized to acquire the infrared spectrum corresponding to the chemical fingerprint of samples. It generates an infrared spectrum by detecting and quantifying the vibrational bond between functional groups, thereby disclosing the entire chemical characteristics of its analytes. Figure 1 shows the conventional FT-IR spectra of flowers, seeds, and leaves of *C. nitidissima*. It can be noticed that the spectra of flowers, seeds, and leaves of *C. nitidissima* have some variances in shape and intensity, particularly after 1800 cm^−1^. The assignments of the peaks and their possible compounds are presented in Table 1.

**TABLE 1:** Peak assignments on the conventional FT-IR spectra of flower, seed, and leaf of *Camellia nitidissima.*

Peak			Primary assignment	Possible compound
Flower	Seed	Leaf		
3391	3400	3420	O-H, ν	Various
2930	2927	2922	C-H, ν_as_	Alkane
	2856	2853	C-H, ν_s_	Alkane
1736	1743	1729	C=O, ν	Ester
	1648		N-H, _δ_	Amide I
1626 and 1629		1624	C=O	Oxalate/aromatic amine
	1548		N-H, _δ_	Amide II
1443	1459		C-H, _δ_	Alkane
1417			C-H, _δ_	Alkene
		1318	C-O, ν	Saccharides
1145	1159	1156	C-O, ν	Saccharides
1102		1098	C-O, ν	Saccharides
	1079		C-O, ν	Saccharides
1058	1046	1050	C-O, ν	Saccharides
	1019		C-O, ν	Saccharides

According to Figure 1’s infrared spectra, the flowers, seeds, and leaves of *C*. *nitidissima* exhibited absorption peaks within the range of 3500–3300 cm^−1^, with an absorption peak at 2925 cm^−1^ attributed to the detection of stretching vibration of O-H groups and asymmetric C-H stretching. A symmetric C-H stretching absorption peak of about 2854 cm^−1^ was also found in the spectra of the seeds and leaves. By examining the spectra, it can be detected that seed samples have a greater absorption peak at 2927 cm^−1^, which postulates that seeds possess a greater amount of asymmetric C-H bonds than flower and leaf samples.

As seen in Figure 1, the seed of *C. nitidissima* contains a large amount of lipid, but no lipid is detected in the flower and leaf. The absorption peaks at 1460 and 1376 cm^−1^ displayed in the seed spectrum could be assigned as the bending mode of the C-H group (Fang et al., 2012; Hofko et al., 2018). The peaks at 1743 cm^−1^ and 1417 cm^−1^, respectively, represent the ester carbonyl C=O group stretching and C-H bending of the unsaturated fatty acid chain. These are classic spectra with a high lipid content (Sun S. et al., 2010). The portrayal of absorption at 1648 and 1548 cm^−1^ in the seed spectra corresponds to the N-H bending and symmetric stretching of N-H groups, respectively (Sun et al., 2011). This suggests that in addition to lipids, seeds contain amide I and amide II proteins. The absorption peaks at 1624 cm^−1^, 1317 cm^−1^, 781 cm^−1^, 661 cm^−1^, and 518 cm^−1^ were observed in the spectrum of the leaf samples. These prominent peaks are attributed to calcium oxalate. The peak at 1317 cm^−1^ might be due to the asymmetric stretching of C=O groups within the oxalate ion (Sun et al., 2011). The peaks at 1317 cm^−1^, 781 cm^−1^, 663 cm^−1^, and 518 cm^−1^ contribute to a strong peak at 1624 cm^−1^, which could also be attributed to asymmetric stretching of C=O groups.

The absorption peaks within the range 1200–950 cm^−1^ are the attributes of various C-O stretches in saccharides and glycosides. *C. nitidissima* seeds had the highest saccharide concentration of the three parts. The absorption peaks at 1159 cm^−1^, 1080 cm^−1^, 1019 cm^−1^, and 999 cm^−1^ were characteristic absorption peaks found in the infrared spectrum of starch, together with the peaks below 950 cm^−1^, showed at 930 cm^−^, 852 cm^−1^, 766 cm^−1^, 576 cm^−1^, and 530 cm^−1^ (Sun S. Q. et al., 2010; Li and Ren, 2011). The occurrence of the highest peaks between 1300–950 cm^−1^ allows us to confidently infer that saccharide, followed by lipid, was the predominant chemical constituent of the seed. Likewise, the major component in the flowers of *C. nitidissima* was saccharides. The highest absorption peaks were found at 1059 cm^−1^, 1103 cm^−1^, and 1145 cm^−1^. By observing the spectrum of leaves, the absorption peaks appeared at 1317 cm^−1^, 1157 cm^−1^, 1098 cm^−1^, and 1051 cm^−-1^, indicating the presence of cellulose (Li and Ren, 2011). Future research should be attempted to confirm the presence of these components.

### Differentiation by SD-IR

In some circumstances, neighboring absorption peaks in the original spectrum overlap. Consequently, derivatives were used to separate these overlapping peaks. Second-derivative infrared spectroscopy (SD-IR) is the tool for quantifying the second derivative of the spectrum in terms of its frequency or wavelength (Whitbeck, 2016). It permits the detection of weak and adjacent absorption spectra that could not be resolved in the original spectrum, thus increasing the specificity of absorption peaks for particular components (Rieppo et al., 2012).

**FIGURE 2:**
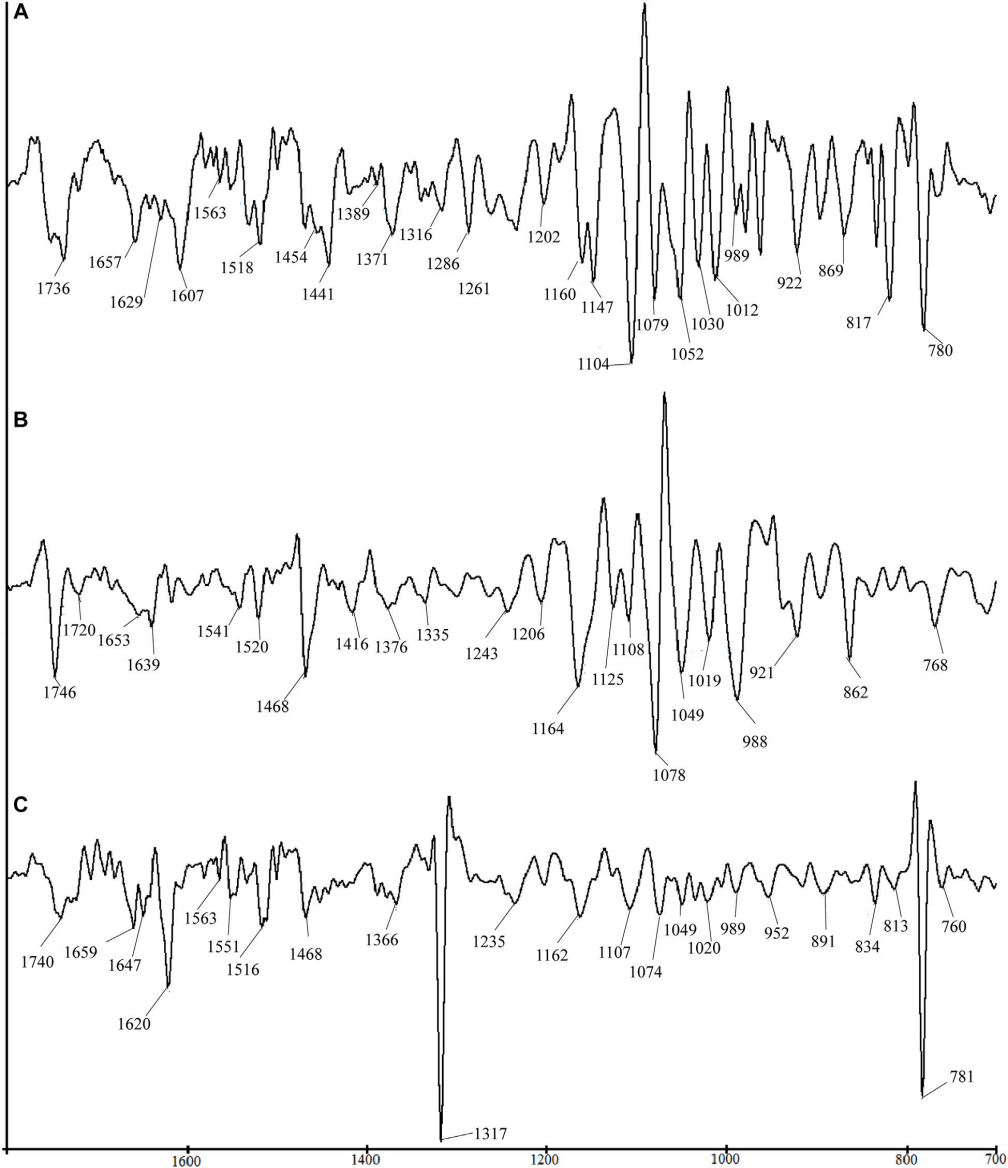
Comparison of SD-IR spectra for different parts of *Camellia nitidissima*: **(A)** flower, **(B)** seed, and **(C)** leaf.

Figure 2 shows the SD-IR spectra of flowers, seeds, and leaves of *C. nitidissima* in the range of 1800–700 cm^−1^. This range contains the major absorption peaks of the analytes’ chemical components. The SD-IR reinforced that the seeds contain a high amount of lipid. Prominent peaks at 1746 and 1468 cm^−1^ can explain this, which also can be seen in the leaves’ SD-IR spectra. However, the lower intensities peaking at 1746 and 1468 cm^−1^, respectively, revealed that leaf and flower samples indeed contain lipids, albeit in a less amount than seeds. Certain facets of the SD-IR spectrum can be used to precisely discriminate between seeds, leaves, and flower samples. Apart from the high lipid content, peaks at 1653 and 1541 cm^−1^ in the seeds exist. These peaks were observed in the SD-IR spectrum of seed and belonged to the stretching vibration of C-O in amide I proteins and the inner bending of N-H in amide II proteins (Sun et al., 2011). These results demonstrated that *C. nitidissima* seeds may contain lipids and proteins.

The SD-IR spectra of the flowers revealed increased absorption peaks that were not seen in the original FT-IR spectrum. The absorption peaks at 1160 cm^−1^, 1149 cm^−1^, 1104 cm^−1^, 1079 cm^−1^, 1051 cm^−1^, 1012 cm^−1^, 989 cm^−1^, 922 cm^−1^, 869 cm^−1^, and 764 cm^−1^ were found in the SD-IR spectrum of flowers. These peaks are comparable to the absorption peaks displayed in the spectra of sucrose (Sun et al., 2011). This proved without the need for a shadow of a doubt that the saccharide composition of *C. nitidissima* flowers is sucrose. Several evaluations of the scientific literature indicate that the *C. nitidissima* flower contains several flavonoids (Jiang et al., 2021). This is proved by the flower’s SD-IR spectra, which confirm flavonoids’ presence. The absorption peaks appeared at 1607 cm^−1^, 1660 cm^−1^, 1575 cm^−1^, 1563 cm^−1^, 1518 cm^−1^, 1454 cm^−1^, 1389 cm^−1^, 1316 cm^−1^, 1261 cm^−1^, 1202 cm^−1^, 1160 cm^−1^, 1079 cm^−1^, 1026 cm^−1^, 1012 cm^−1^, and 1009 cm^−1^ resemble the characteristic peaks of flavonoid (Heneczkowski et al., 2001; Catauro et al., 2015; Selestin Raja et al., 2018).

### Differentiation by 2D-IR

In contrast to conventional FT-IR, two-dimensional correlation infrared (2D-IR) spectroscopy was generated by introducing external perturbation such as heat (Sun S. et al., 2010). Different functional groups or components react differently to an external disturbance, enabling the projection of additional information inaccessible with conventional FT-IR or SD-IR. In other words, when similar external perturbation is applied, 2D-IR enhances spectral resolution and offers additional information based on the inter- and intra-molecular responses between distinct functional groups (Ch’ng et al., 2016).

The asynchronous 2D-IR correlation spectrum consists of two peak types: auto-peaks and cross-peaks. Auto-peaks are displayed diagonally, and their intensity corresponds to the auto-correction of spectral intensity fluctuations brought on by external disturbances. The cross-peaks are offset from the diagonal and reflect the related variations in spectral intensity measured at two distinct wave numbers. A prominent cross-peak will be apparent if the absorption intensities are comparable (Yu et al., 2005). A positive cross-peak (red or green area) indicates an externally induced change in the peak intensities of two distinct groups in the same direction. In contrast, a negative cross-peak (blue area) is produced when coordinated variations in peak intensities occur in opposite directions. Cross-peak will not be detected if IR intensity variations are not coordinated (Tan et al., 2018).

**FIGURE 3:**
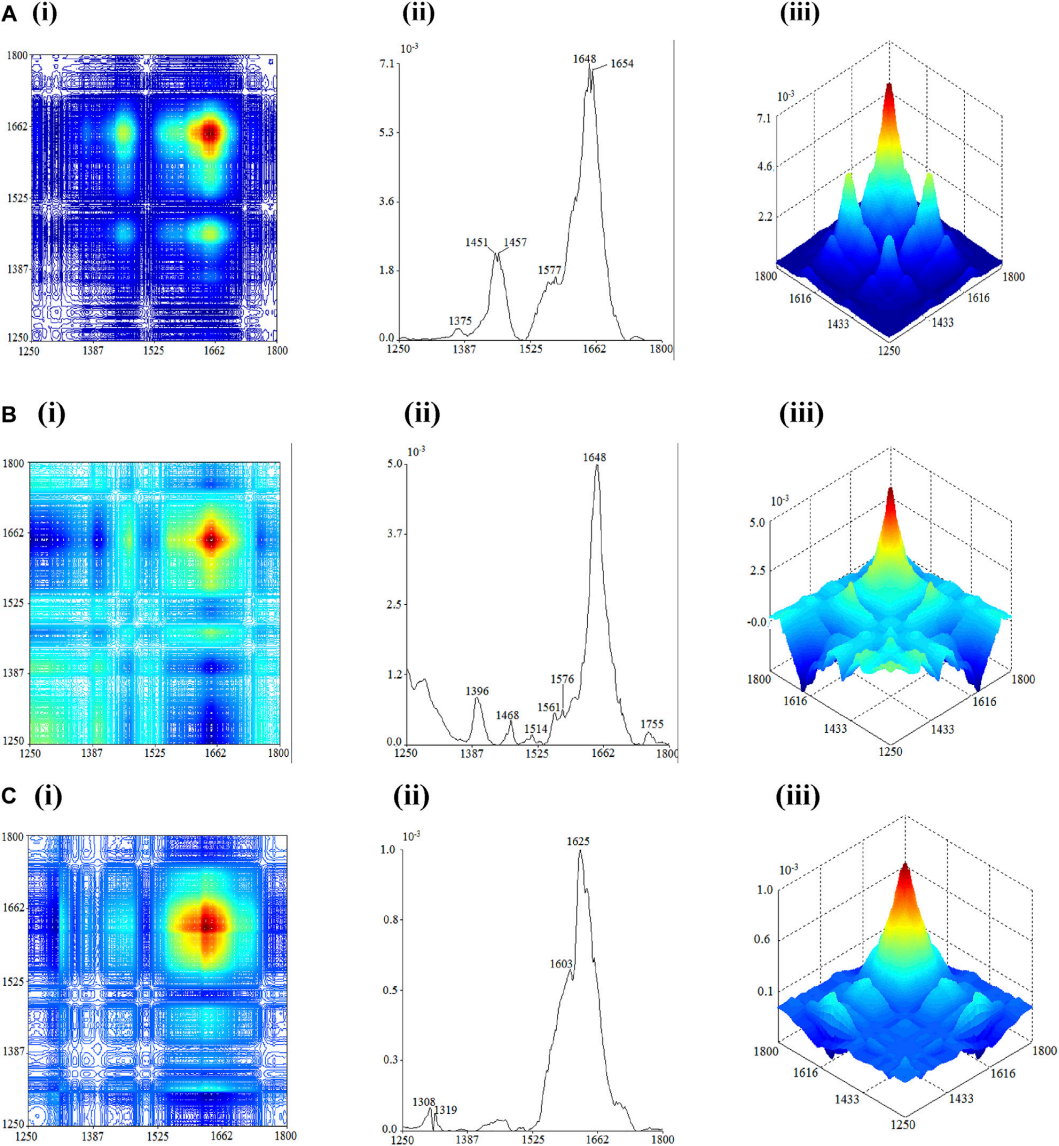
2D-correlation IR spectra of each part of *Camellia nitidissima* in the range of 1800–1250 cm^−1^: **(A)** flower, **(B)** seed, and **(C)** leaf.

The 2D-IR results of the flowers, seeds, and leaves of *C. nitidissima* within the wavenumber of 1250 cm^1^ to –1800 cm^−1^ is shown in Figure 3. The 2D-IR of both the flower and seed showed the strongest auto-peak at 1648 cm^−1^, but the positions of other main auto-peak of the flower and leaf samples differed. The main auto-peak of flowers can be observed around 1451 cm^−1^, 1457 cm^−1^, 1648 cm^−1^, and 1654 cm^−1^, and they have positive corresponding cross-peaks. Weak auto-peaks were observed at 1375 cm^−1^, 1577 cm^−1^, and 1744 cm^−1^. The auto-peaks showed at 1375 cm^−1^, 1451 cm^−1^, 1457 cm^−1^, 1648 cm^−1^, and 1744 cm^−1^ corresponded to the absorption peaks of flavonoids (Choong, 2016). These provide further justification that flowers of *C. nitidissima* contain flavonoid compounds.

The 2D-IR analysis of seeds revealed auto-peaks between 1600 and 500 cm^−1^ and a strong peak at 1648 cm^−1^, with positive cross-peaks appearing between them. These undoubtedly corresponded to variations in amide I and amide II proteins induced by heat exposure. Once again, it was discovered that *C. nitidissima* seeds contain protein compounds. The auto-peak found at 1468 cm^−1^ reflects the absorption peak of C-H bending in lipid (Sun S. et al., 2010; Li and Ren, 2011). The positive cross-peak formed between 1468 cm^−1^ with the auto-peaks in the range of 1600–500 cm^−1^ and 1648 cm^−1^ can be characterized by the lipids and proteins in the seed having comparable thermal stability. These traits distinguish the seeds of *C*. *nitidissima* from the plant’s flowers and leaves.

The 2D-IR spectra of *C. nitidissima* leaves contained peaks between 1250 and 1800 cm^−1^ and were notably different from the spectrum of other plant parts, with only four auto-peaks located at 1308 cm^−1^, 1319 cm^−1^, and 1603 cm^−1^ and the highest peak located at 1625 cm^−1^. The presence of auto-peaks at 1319 cm^−1^ and 1625 cm^−1^ corresponds to the absorption peaks of calcium oxalate, which only appear in the 2D-IR spectra of leaves. Both auto-peaks at 1319 cm^−-1^ and 1625 cm^−1^ form positive cross-peak but form negative cross-peak at 1308 cm^−1^. These are the key characteristics in the spectrum of calcium oxalate (Li and Ren, 2011; Sun et al., 2011).

**FIGURE 4:**
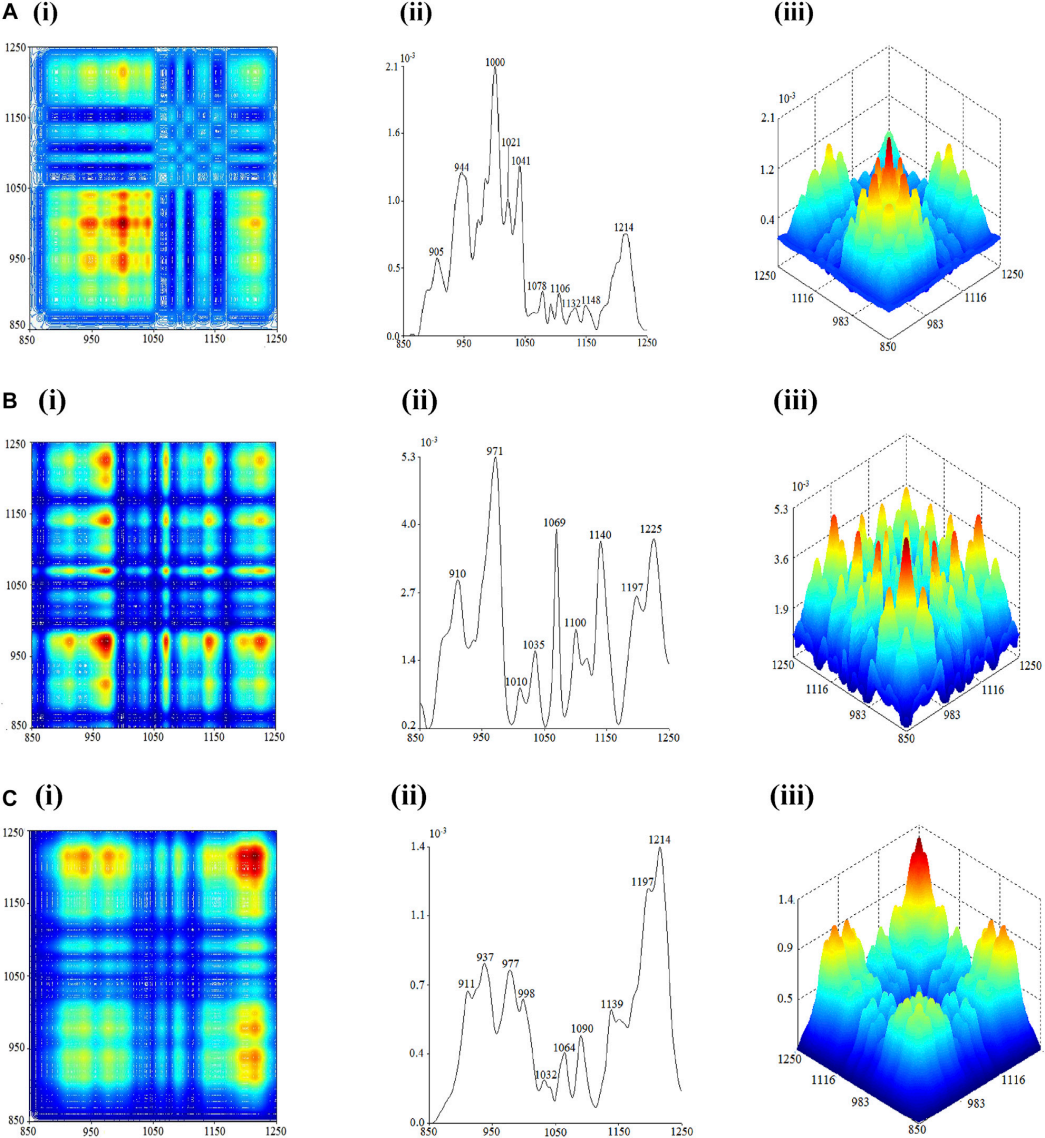
2D-correlation IR spectra of each part of *Camellia nitidissima* in the range of 1250–850 cm^−1^: **(A)** flower, **(B)** seed, and **(C)** leaf.

Within the range of 1250–850 cm^−1^, the strongest auto-peaks of flowers, seeds, and leaves of *C. nitidissima* were located at 1000 cm^−1^, 971 cm^−1^, and 1214 cm^−1^, respectively. Thus, the outcome of this 2D-IR spectrum can be used as one of the discriminating characteristics of various *C*. *nitidissima* portions. The 2D-IR spectrum of the flower is shown in Figure 4A. The auto-peaks appear at 1132 cm^−1^, 1106 cm^−1^, 1078 cm^−1^, 1041 cm^−1^, 1021 cm^−1^, 1000 cm^−1^, 944 cm^−1^, and 905 cm^−1^. Between these auto-peaks, positive cross-peaks have formed. These indicators point to the existence of sucrose in flowers (Li and Ren, 2011).

The 2D-IR spectra of the seed and leaf are in the range of 1800 cm^−1^–1250 cm^−1^ and are quite similar. Both carry auto-peaks at 910 cm^−1^, 971 cm^−1^, 1035 cm^−1^, 1069 cm^−1^, 1140 cm^−1^, and 1197 cm^−1^. There were positive cross-peaks formed between each other. The auto-peaks at 937 cm^−1^, 998 cm^−1^, 1090 cm^−1^, and 1214 cm^−1^ were the key features in differentiating the leaves from the seeds in the 2D-IR spectrum. In addition, the seed spectrum contains auto-peaks at 1010 cm^−1^, 1100 cm^−1^, and 1225 cm^−1^ that are absent in the leaf spectrum, which is another distinguishing trait of *C. nitidissima* leaves and seeds. Based on the 2D-IR spectrum of seeds, it can be confirmed that there is a presence of starch content in seeds upon detection of auto-peaks at 910 cm^−1^, 971 cm^−1^, 1069 cm^−1^, 1140 cm^−1^, and 1225 cm^−1^, which resemble auto-peaks found in starch (Sun S. et al., 2010). The absence of auto-peak at 1225 cm^−1^ indicates that the leaves do not carry starch as their saccharide content.

### Differentiation by the chemometric technique

Chemometric approaches were used to further distinguish between the flowers, seeds, and leaves of *C. nitidissima*. Chemometric is a method for extracting information from chemographic data. In recent years, increased computer technology has made chemometric analysis the primary technique in analysis studies, owing to its ability to reduce the time required to conduct a valid analysis. PCA is a statistical technique that uses an orthogonal transformation to minimize the dimensionality of multivariate data while maintaining as much information as feasible. The PCA-class is utilized to convert and organize the data depending on specific classes or variables. It provides graphical overviews and investigates the general sample distribution trend for outliers, aggregation, and dispersion through graphical representations (Jolliffe and Cadima, 2016; Li and Liu, 2019). Partial least square (PLS) regression is a multivariate method for evaluating the relationship between a descriptor matrix X and a response matrix Y. Partial least square discriminant analysis (PLS-DA) is an algorithm evolved from PLS.

PLS-DA identifies variable selection in addition to predictive and descriptive models (Lee et al., 2018). PLS-DA differentiates more clearly than PCA because it acquires information by decomposing both X and Y matrices, whereas PCA only decomposes the X matrix (Wu et al., 2018). Orthogonal partial least square (OPLS) is an extension of PLS regression. It breaks down the X matrix into two parts that correlate the orthogonal to the Y matrix (Bylesjö et al., 2006). This will provide a better analysis as the composition of the orthogonal variation increases (Trygg and Wold, 2002; Bylesjö et al., 2006). Analogous to PLS-DA, it can be used for precise distinction analysis (OPLS-DA) (Cloarec et al., 2005).

**FIGURE 5:**
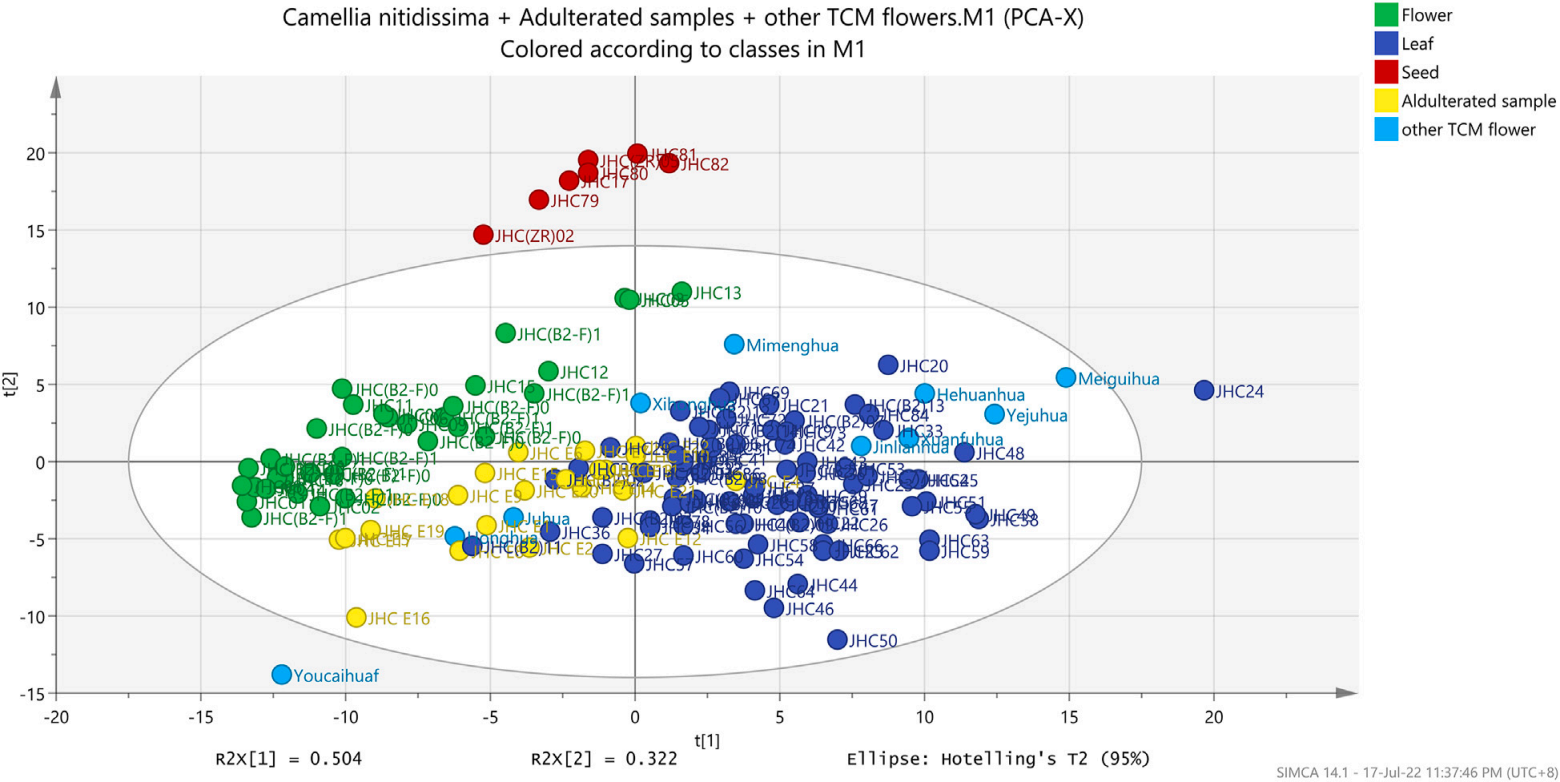
Unsupervised PCA score plot of different parts of *Camellia nitidissima* and other flower type TCM Materia Medica.

**FIGURE 6:**
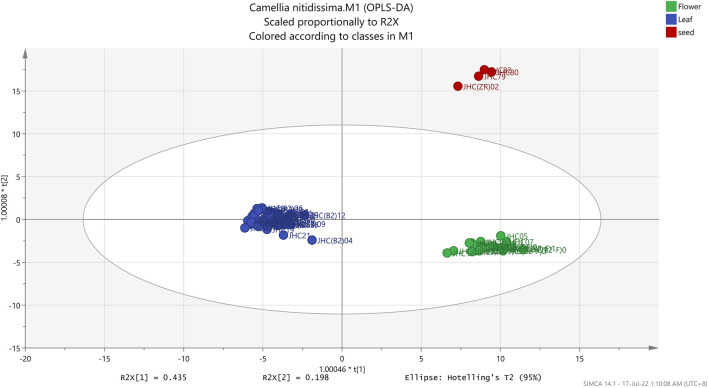
OPLS-DA score plot of different parts of *Camellia nitidissima*.

Using the FT-IR spectra, this study used PCA, PCA-class, and OPLS-DA to detect the differences between three parts of *C*. *nitidissima* (Figures 5, 6). Unsupervised PCA was used to obtain a preliminary overview of the 141 samples (Figure 5). As illustrated in Figure 5, the samples were divided into three groups based on their two initial principal components of 73.5115%: flower, leaf-TCM flower reference standard-mixture samples, and seed. In addition, PCA-class showed that the values of R^2^X and Q^2^ for flower, leaf, and seed groups were 0.899–0.991 and 0.773–0.967, respectively. PCA-class’s sensitivity, accuracy, and specificity for different parts of *C. nitidissima* were all >94%. Furthermore, PCA-class study discriminated 27 out of 32 samples (adulterated *C. nitidissima* and TCM reference flower samples) from the *C. nitidissima* flower group.

In OPLS-DA analysis, R^2^Y (goodness of fit) and Q^2^Y (goodness of predictability) values were used to evaluate the OPLS-DA model (Lee et al., 2017). The R^2^Y and Q^2^Y values fall between 0 and 1.0, and the higher R^2^Y value deduces a better model fit. The Q^2^Y values within the range of 0.5–0.9 indicated good predictability, while those below 0.9–1.0 were considered excellent predictability. The model validation uses the R^2^Y and Q^2^Y intercepts obtained in the permutation test. In a valid OPLS-DA model, the R^2^Y and Q^2^Y values must be lower than 0.3 and 0.05, respectively.

**FIGURE 7:**
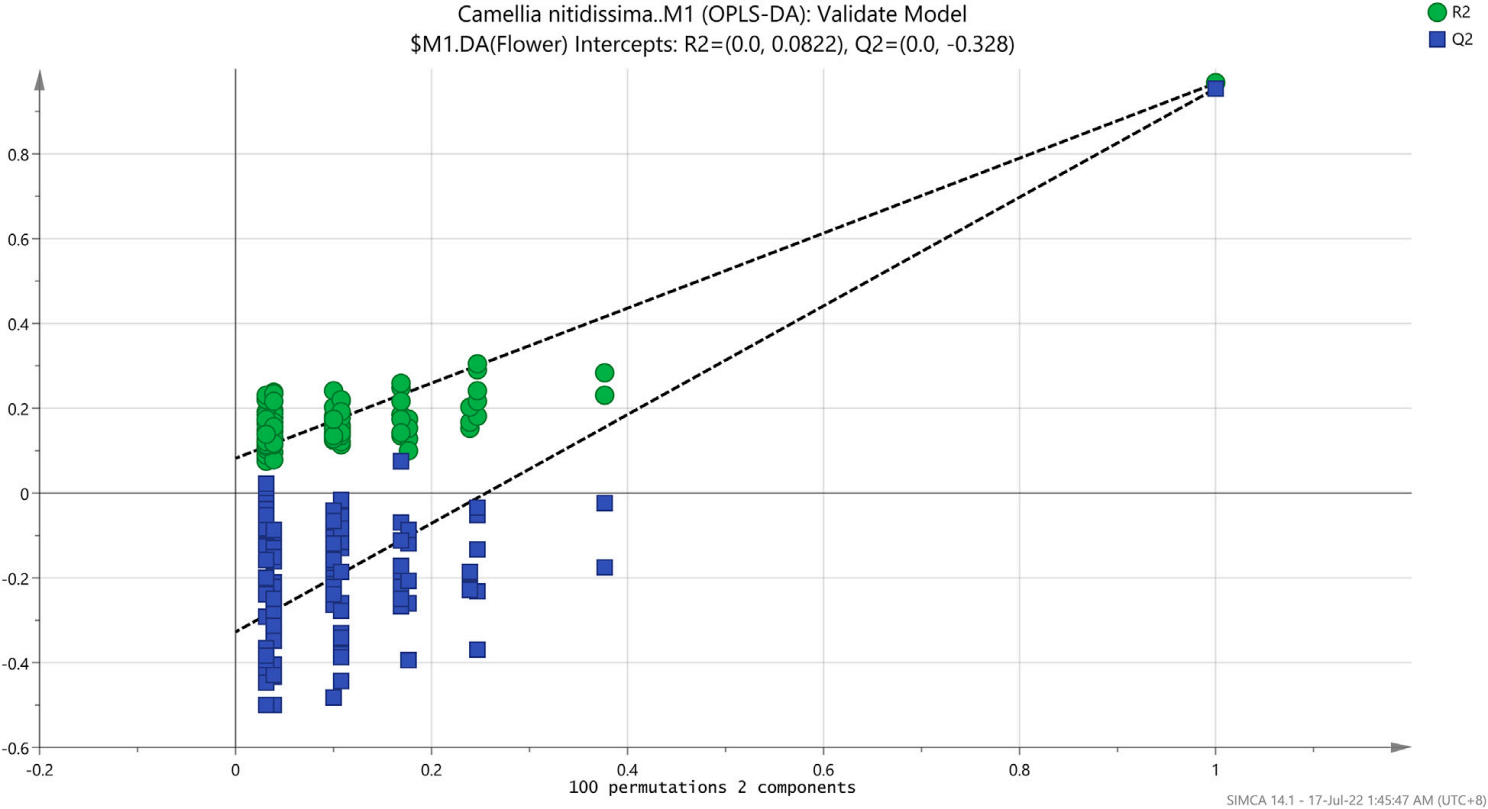
Permutation test of the OPLS-DA model.

The root-mean-squared error of estimation (RMSEE), root-mean-squared error of prediction (RMSEP), and root-mean-squared error of cross-validation (RMSECV) values were further used to evaluate the OPLS-DA model of both data sets. Similar to R^2^ and Q^2^, the values for these parameters ranged from 0 to 1.0. The smaller the RMSEE and RMSEP values, the higher the predictability and accuracy of the model (Lee et al., 2017). For RMSECV, smaller values mean lesser variables such as noise removal (Wu et al., 2018). According to Table 2, the RMSEE, RMSEP, and RMSECV values for the model generated by the FT-IR spectrum were 0.08615, 0.09617, and 0.2226, respectively. With low values in all three analyses, the OPLS-DA model using data sets has demonstrated great accuracy and predictability. The R^2^Y and Q^2^Y intercepts for the 100-repetition permutation test were 0.0822 and -0.328, respectively (Figure 7).

**TABLE 2:** Parameters of PCA, PCA-class, and OPLS-DA model.

Chemometric model	Part	R^2^X	R^2^Y	Q^2^Y	R^2^Y intercept (permutation)	Q^2^Y intercept (permutation)	RMSEE	RMSE_CV_	RMSEP
PCA	--	0.991	--	0.978	--	--	--	--	--
	Flower	0.991		0.956	--	--	--	--	--
PCA-class	Leaf	0.991		0.967	--	--	--	--	--
	Seed	0.899		0.773	--	--	--	--	--
OPLS-DA	--	0.961	0.968	0.957	0.082	-0.328	0.08615	0.09617	0.2226

In multivariate data analysis, sensitivity, specificity, and accuracy were used broadly to represent a test’s precision. A test can be specific but not sensitive, or vice versa (Ma et al., 2016). Both sensitivity and specificity are equally important. A good multivariate test showed high sensitivity and specificity. Sensitivity indicated how well the test detected samples with the specified condition, whereas specificity indicated how well the test identified the samples without the given condition (Li and He, 2018). Similar to sensitivity and specificity, another statistical metric, accuracy, is frequently paired with sensitivity and specificity. Accuracy assesses how well a test classifies, identifies, and excludes samples, thus assessing the test’s precision. A formula can be used to describe sensitivity, specificity, and precision (Ma et al., 2016; Trevethan, 2017; Li and He, 2018). In this experiment, the sensitivity, specificity, and accuracy of the OPLS-DA model were determined, indicated with values of 100% for all three parameters. The model correctly classified all the samples into flowers, leaves, and seeds. Furthermore, the OPLS-DA study was able to discriminate 26 out of 32 samples (adulterated *C. nitidissima* and TCM reference flower samples) from the *C. nitidissima* flower group.

## Conclusion

This study revealed that different portions of the same plant, *C. nitidissima*, contain distinct major constituents. The operational technique for identifying and differentiating the elements of a plant—the flowers, seeds, and leaves—produced a blueprint for future studies. This research demonstrated the dependability and rapidness of multi-step infrared and chemometric analysis to identify sample chemical components. Chemical fingerprinting, including peak shape, position, and intensities, gave unique markers for detecting chemical compositions, thus relating to the separation of *C. nitidissima* plant segments. This is crucial in the study of herbs because it allows for the rapid and thorough evaluation of samples and offers the data needed to be zero in the components of the herbs that have a specific pharmacological effect on the human body. Although this study established a clear differentiation between the chemical composition of *C. nitidissima* flowers, leaves, and seeds, additional research is needed to determine *C. nitidissima’s* whole chemical composition.

## Data Availability

The original contributions presented in the study are included in the article/Supplementary Material; further inquiries can be directed to the corresponding authors.
